# Chronic myeloid leukemia therapy in the era of tyrosine kinase inhibitors. The first molecular targeted treatment

**Published:** 2010-05-25

**Authors:** H Bumbea, AM Vladareanu, D Cisleanu, L Barsan, M Onisai, I Voican

**Affiliations:** * ‘Carol Davila’ University of Medicine and Pharmacy. Emergency University Hospital Department of HematologyRomania; ** Emergency University Hospital Department of Hematology Romania

**Keywords:** chronic myeloid leukemia, tyrosine kinase inhibitors, molecular target, BCR–ABL translocation

## Abstract

Chronic Myeloid Leukemia is the first malignant disorder with a specific genetic abnormality in the background. Known as a disease with an inexorable progression to acute leukemia for many years, its natural history has been dramatically improved by the use of tyrosine kinase inhibitors (TKI). They represent the first molecular targeted therapy addressed to a neoplastic disorder. From these new classes of drugs, Imatinib was the first drug ever used, and it remains the standard therapy for patients in chronic phase with CML, having a global survival of 86%, for 7 years. The 2^nd^ generation of TKI (Dasatinib, Nilotinib) is indicated for the patients who are refractory or intolerant to Imatinib. The other TKI have good promises to be efficient on the mutations of BCR–ABL transcript, especially to non–responsive T315I mutation.

The new era of molecular target therapy is a new hope of life for all cancer patients.

## Background

Chronic Myeloid Leukemia (CML) is a myeloproliferative disorder due to a clonal pluripotent stem cell disorder. Wirchow and Binnet described it for the first time in 1845 and it was the first malignant disease with a genetic marker involved in its etiology. The genetic marker is represented by the Philadelphia chromosome (Ph) described in 1960 and the results from a reciprocal exchange of material between two chromosomes: 9 and 22 chromosomes, t(9;22)(q34;q11)[1]. The Philadelphia chromosome is identified in over 95% of patients with CML and represents the genetic hallmark of CML; the molecular marker is the presence of BCR–ABL fusion gene – mandatory for positive diagnosis.[2]

## Pathogeny

**Figure 1:**
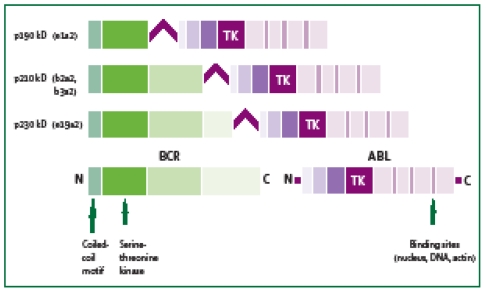
Graphical representation of BCR–ABL transcripts resulting from the translocation t(9;22)

CML is a hematopoietic stem cell disorder, developed from the translocation t(9;22)(q34;q11), known as Philadelphia chromosome. This translocation produces the juxtaposition of ABL gene on chromosome 9 with BCR gene from chromosome 22, resulting in the fusion gene, which encodes the BCR–ABL transcript and the fusion proteins with abnormal tyrosine kinase activity [2] (Figure 1). CML pathogeny is well known, and it has been studied in detail at a molecular level, but the mechanism of translocation is not very well understood. Exposure to radiation is suggested as a possible cause, because of the increase in incidence after the nuclear explosions from Hiroshima and Nagasaki.[3]

BCR–ABL fusion gene produced from BCR and ABL genes, usually encoded the protein p210 with tyrosine kinase activity. This activity is responsible for the proliferation mechanisms in CML. There are two fusion genes, which are described as having different molecular weight, p190, specific for acute lymphoblastic leukemia, and rarely, p230.[3]

## Diagnosis

In most cases, CML is diagnosed by a specific hematological picture of peripheral blood, with excessive granulopoiesis on left shift. Diagnosis criteria established by last ESMO recommendations are:

- Leucocytosis +/–Thrombocytosis (150–450 x 10^9^/l)
- Left shift of differential–to myeloblasts
- Basophils < 20%
- Splenomegaly (>50%)
- Ph1 chromosome (t(9;22)/ BCR–ABL fusion gene in peripheral blood/bone marrow detected by cytogenetic/PCR analysis

In about 5% of cases, Ph1 chromosome is absent, and the diagnosis is confirmed by BCR–ABL transcript detection through FISH or PCR.[1]

## Treatment

**Figure 2:**
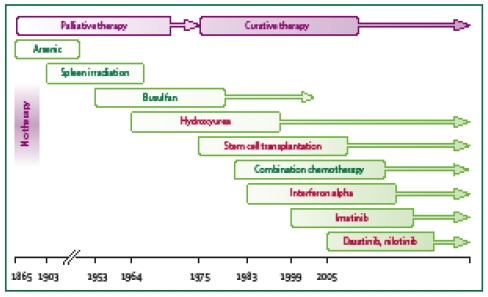
Graphical representation of treatment options in CML

For many years, the specific treatment for CML consisted of cytoreduction, and the combination between immunomodulatory (interpheron–alpha) and Ara–C represented an important change in CML patients' prognosis in the middle of '90s (Figure 2).

**Figure 3:**
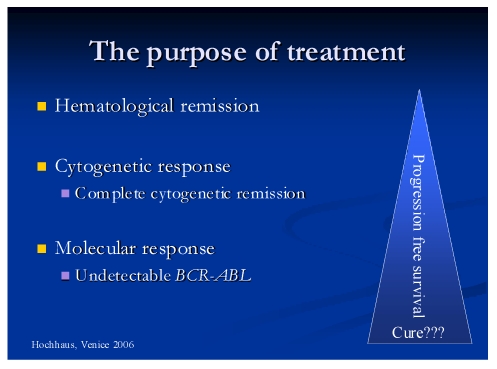
Graphical representation of treatment purpose in CML

Tyrosine kinase inhibitors discovery at the end of the millennium represented a crucial moment in the treatment of CML. The purpose of the treatment in CML is to obtain three complete responses: hematological, cytogenetically, molecular (Figure 3).

The mechanism of action of TKI is accomplished by blocking the locus with a TK function at the BCR–ABL transcript, thus representing the first treatment, which specifically inhibits a genetic alteration as the etiology of malignant process.

TKI are classified according to the target in BCR–ABL transcript, as it follows:

- abl TK inhibitors
  - Imatinib (Novartis)
  - Nilotinib (AMN107, Novartis)
- Dual Abl/Src inhibitors
  - Dasatinib (BMS 254825, Bristol–Myers Squibb)
  - SKI–606 – ‘bosutinib’ (Wyeth)
  - AP23464 (Ariad Pharmaceuticals)
  - AZD0530 (Astra–Zeneca)
- Dual Abl/Lyn inhibitor
  - NS–187 (INNO–406) (Nippon–Shinyaku)
- Non–ATP–binding inhibitors active against T315I
  - ON 012380 (Onconova)
  - VX–680 (Aurora kinase inhibitor) a Merck 0457–T315I
  - SGX–70430 (SGX Pharma)
  - GNF–2 (Genomics Novartis Foundation)

**Imatinib** was the first inhibitor discovered for tyrosine kinase and it remains the standard treatment in CML. It is an ABL specific tyrosine kinase inhibitor, which inhibits the proliferation of CML cell lines by inhibiting ATP binding at ABL tyrosine kinase.

**Figure 4:**
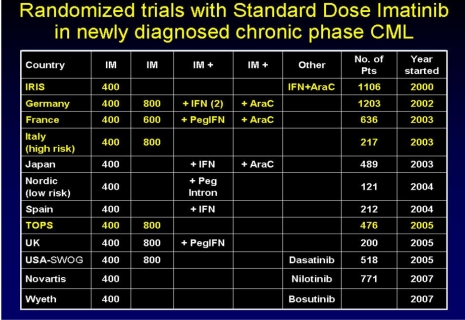
Comparative studies with Imatinib in CML

Many comparative studies have been reported since the beginning of the use of Imatinib in patients with CML. They compared the efficacy of Imatinib with other therapies such as Interferon alpha, cytarabine, stem cell transplantation (Figure 4).

The **IRIS study** compared the efficacy of Imatinib (in doses of 400, 600, 800 mg/day) to alpha–INF + ARA–C, in a follow–up period of 6–7 years. 553 patients were randomized in each branch. The IRIS study estimated adverse reactions rates, complete hematological, cytogenetic and molecular responses rates, progression free survival to accelerated phase or blast crisis CML .[4]

The conclusions of the IRIS study were the following: 82% of Imatinib treated patients achieved complete cytogenetic response; only 17% of them relapsed and 3% progressed to accelerated phase / blastic crisis; 2% resulted in death. There was a long–term response, and annual risk of progression is decreasing in time.

The molecular response rate was significant, and survival for 7 years was of 86%, superior to any other therapy.

Another important trial was **SPIRIT**, from the French Working Group in CML, on a group of 636 patients, on one–year follow–up period. The study compares the response to Imatinib in doses of 400 mg versus 600 mg versus Imatinib and another therapy (Ara–C or IFN–alpha). The common conclusion of these studies was that Imatinib alone or in combination with other therapies is the first line treatment in patients with chronic phase CML.

## Resistance to Imatinib

There is a category of CML patients, who do not respond to standard dose of Imatinib, or, they relapse after an initial response. Those patients are considered resistant to Imatinib. Drug resistance is associated with the reactivation of BCR–ABL signal transduction of those patients who start Imatinib in the early chronic, late chronic and accelerated phase of CML; 12%, 32%, and respectively 62% of CML patients in early chronic, late chronic and accelerated phase develop resistance mutations within 2 years from the first administration of the treatment. The resistance is based on multiple mechanisms, but the most important one is related to the mutations in BCR–ABL gene. 

**Figure 5:**
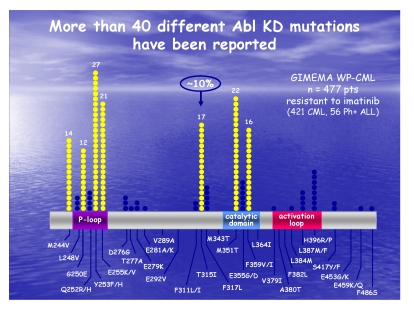
Graphical representation of BCR–ABL mutations and their sensitivity to different ITK

The mutation analysis is vital to the selection of the proper therapeutic option for Imatinib resistant patients (Figure 5). The detection of new mutations suggests genetic instability during disease progression. The therapeutic options for Imatinib resistant patients consist either of an increased Imatinib dosage to 600mg or 800mg [5] , or of the Imatinib replacing with the 2^nd^ generation TKI – Dasatinib or Nilotinib [6]. Both 2 ^nd^ generation TKI are recommended for CML patients resistant or intolerant to Imatinib.

**Nilotinib** is a selective tyrosine kinase inhibitor with an inhibiting effect thirty times higher than Imatinib. It is approved for the treatment of patients with CML in chronic or accelerated phase and for patients who are intolerant to Imatinib.

**Dasatinib** is more efficient in chronic phases in CML patients after the failure of Imatinib, than in accelerated phases or blast crises. Dasatinib versus Imatinib 800 mg daily in treatment of adults with CML chronic, accelerated or blast crises, with resistance to Imatinib in standard doses (400–600 mg daily), achieved a better cytogenetic and molecular response and a longer time of free disease survival. Dasatinib is currently indicated in a dosage of 100mg daily, having the same effects as doses of 70mg twice daily, but with less adverse reactions. 

The lack of crossover adverse reactions with Imatinib and the small number of adverse reactions, indicates Dasatinib in intolerance to Imatinib patients. The present indication of Dasatinib is in the treatment of CML patients with resistance or intolerance to Imatinib [7].

The new generations of TKI are still in trials, but with good promises especially from the TKI against T315I mutation.

## Conclusions

Chronic myeloid leukemia represents the first malignancy with documented molecular etiology. Tyrosine kinase inhibitors opened a new era not only in the CML treatment, but also in the treatment of other malignant disorders. These drugs are a new hope for life in cancer patients. TKI dramatically changed the outcome of a malignant hematological disorder with an inexorable progression to acute leukemia, and it represents the first model of molecular target therapy. The tyrosine kinase inhibitors represent a new era not only in CML, but also in the therapy of other hematological or non–hematological neoplasms. They are a new hope of life for all the cancer patients.
